# New species of *Cystolepiota* from China

**DOI:** 10.1080/21501203.2016.1239231

**Published:** 2016-10-19

**Authors:** Meng-Lin Xu, Guo-Jie Li, Jun-Liang Zhou, Xu-Ming Bai, Rui-Lin Zhao

**Affiliations:** a State Key Laboratory of Mycology, Institute of Microbiology, Chinese Academy of Sciences, Beijing100101, China; b Institute of Microbiology and Beijing Key Laboratory for Forest Pest Control, Beijing Forestry University, Beijing100083, China

**Keywords:** Agaricaceae, ITS, phylogenetics, taxonomy

## Abstract

In this paper, a new species, *Cystolepiota pseudofumosifolia*, is introduced. *C*. *pseudofumosifolia* is characterized by granulose or powdery pileus with an anatomic structure that is loosely globose, as well as ellipsoid cells in chains in the pileus covering the cheilocystidia. This new species is compared to the related and similar *Cystolepiota* species in morphology and molecular phylogeny based on Internal transcribed spacer sequences. Both types of data support our specimens as a new species in the genus *Cystolepiota*.

## Introduction

*Cystolepiota* Singer (Agaricaceae/Agaricales) is one genus of lepiotaceous fungi. *Cystolepiota* species are generally saprotrophic, pileus less than 50 mm in diameter, and basdiocarp heavily covered by granules on the pileus and stipe. The edge of the pileus is often appendiculate; an annulus is present but often becomes an annular zone at the stipe; the spore print is white (Vellinga 2004a; Bau and Liu 2010; Gierczyk et al. 2011). Species of *Cystolepiota* are very similar to *Melanophyllum haematospermum* (Bull.: Fr.) Kreisel which is also covered with greyish-brown powder or granules on the pileus and stipe; *Lepiota* sect. *Echinatae* has even been considered as a part of *Cystolepiota* by Knudsen (1978) for its inflated subglobose to ellipsoid hyphal cells of pileipellis. However, some morphological characters, such as the coloured spore print, separate *M. haematospermum* and *L*. sect. *Echinatae* from *Cystolepiota* (Vellinga 2001).

Presently, more than 10 *Cystolepiota* species have been identified and they have been well characterized in Europe and Western North America (Hausknecht and Pidlich-Aigner 2004; Vellinga 2004b, 2006, 2007; Gierczyk et al. 2011). The most complete phylogenetic study of *Cystolepiota* was conducted by Vellinga (2007). In this study, six *Cystolepiota* species were examined, and the monophyly of the genus was supported. This molecular approach also clarified some taxonomic problems, resulting in transferring *Lepiotafumosifolia* Murrill into *Cystolepiota* (Vellinga 2007). Some species have been recorded in Asia, such as *Cystolepiotahetieri* (Boud.) Singer, *Cystolepiotaseminuda* (Lasch) Bon from Iran (Asef & Muradov 2012; Albuquerque et al. 2010), *Cystolepiotapseudogranulosa* (Berk. & Broome) Pegler from Sri Lanka (Pegler 1972), and *Cystolepiotafurfuracea* T.K.A. Kumar & Manim from India (Arun Kumar and Manimohan 2009). In China, only a small number of species have been recorded from this genus, which distributed from the temperate to subtropical regions (Bau and Liu 2010; Chou 2010; Yang et al. 2011).

We conducted mushroom surveys from several national natural reserves in Yunnan Province, southwestern China in 2011 and 2012. Several new species were discovered and this paper presents one new *Cystolepiota* species. Vellinga split the genus of *Cystolepiota* into three groups based on microscopic features, that is, sect. *Cystolepiota*, sect. *Pulverolepiota* (M. Bon) Vellinga, and sect. *Pseudoamyloideae* Singer & Clem (Vellinga 2001). In this paper, a new species belonging to *Cystolepiota* sect. *Cystolepiota* is described, illustrated, and compared with related taxa.

## Materials and methods

### Morphological examination

Collections were made in the field, photographs were taken in situ, and the odour and discoloration after bruising were recorded. The fruiting bodies were wrapped in aluminium foil and kept separately in a compartmented box in order to avoid mixing or crushing (Zhao et al. 2010). The description of macroscopic characters, chemical testing, and further photography of fresh samples were carried out as soon as possible following the methodology described by Largent (1986). Colour terms follow those of the Online Auction Color Chart™ (www.OnlineAuctionColorChart.com). The samples were then dried completely using a drier, sealed in plastic bag, and deposited in the Herbarium of Mycology, Institute of Microbiology, Chinese Academy of Sciences (HMAS), Beijing, China.

Microscopic characters were examined from the pileus, gill, stipe, and veil of dried specimens following the protocols of Largent (1986) and Nauata (2001). First, a small piece of material was softened with absolute ethanol, then sliced by hand and successively mounted in 5% NaOH, Congo red solution, and Cresyl blue for observation under the microscope. The anatomic structures of the lamellae, pileipellis, stipitipellis, and partial veil, including basidiospores, basidia, cystidia, and clamp connections, were examined (Robin 1999). The size of the basidiospores, basidia, and cystidia were observed based on at least 20 measurements with the following abbreviations: avl = average length, avw = average width, Q = quotient of length to width, and avQ = average quotient.

### Molecular phylogenetic analyses

#### DNA extraction, PCR, and sequencing

DNA was extracted from dried specimens using a commercial DNA extraction kit (E.Z.N.A. Forensic Kit, D3591-01, Omega BioTek). The polymerase chain reaction (PCR) reactions and sequencing were performed using the primers ITS5 and ITS4 (White et al. 1990). The PCR reaction contained 5 μL PCR GoTaq buffer (5X, Promega), 2.5 μL deoxy-ribonucleotide triphosphate mix (1.2 mmol/L, Eurobio), 0.5 μL bovine serum albumin (10 mg/mL, Promega), 1 μL of each primer (25 mmol/L), 0.2 μL Taq polymerase (5 U/μL, GoTaq Promega), 1 μL DNA template, and ddH_2_O up to 25 μL. PCR thermal cycling conditions followed those of Zhao et al. (2011) with some modifications. The programme was 5 min at 95°C; 35 cycles (denaturation 1 min at 94°C, annealing 1.5 min at 52°C, extension 1.5 min at 72°C); 5 min at 72°C for the final extension. The PCR products were examined electrophoretically in an agarose gel stained with ethidium bromide (EB), then sequenced by Biomed Co. Ltd., using an ABI 3730XL Analyzer and ABI BigDye 3.1 Cycle Sequencing Kit.

#### Sequence alignment and phylogenetic analyses

Preliminary analysis of the Internal transcribed spacer(ITS) sequences were performed using Basic BLAST (www.blast.ncbi.nlm.nih.gov) in order to detect the contamination of materials. Sequences were downloaded from GenBank, and manually adjusted using Mafft 7.300 and BioEdit 7.0.9.0 when necessary (Hall 1999; Katoh and Standley 2013). Gaps were not removed from the alignment. The sequence alignments were submitted online (www.phylogeny.fr.cgi) to determine the relationship between these sequences.

Maximum parsimony (MP) analyses were performed using PAUP*4b10 (Swofford 2004). Maximum bootstrap (BS) values were obtained from 1000 replicates from the MP analysis. The tree bisection–reconstruction (TBR) algorithm was used in a heuristic search, and BS support was determined with 1000 replicates (Felsenstein 1985). A total of 5000 Maxtrees were set. Branches of zero length were collapsed, and all most parsimonious trees were saved. Clade stability was assessed in a BS analysis with 1000 replicates, each with 10 replicates of a random stepwise addition of taxa.

A Kishino–Hasegawa test (KH test) (Kishino and Hasegawa 1989) was performed to determine whether trees were significantly different. The consistency index (CI), retention index (RI), rescaled consistency (RC) index, homoplasy index (HI), and tree length (TL) were also calculated.

The best nucleotide substitution model for Bayesian analyses was chosen using MrModeltest 2.3 (Nylander 2004). Bayesian phylogenetic inference was performed using MrBayes 3.2.6 (Huelsenbeck and Ronquist 2001; Huelsenbeck et al. 2001; Ronquist and Huelsenbeck 2003). Four Markov chains were run for 1,000,000 generations and sampled every hundredth generation, resulting in 10,000 trees. The trees sampled before the searches reached an average deviation of split frequencies lower than 0.01 were discarded as the burn-in, and the remaining trees were used to calculate Bayesian posterior probabilities (PPs) for the individual clades. The resulting trees were visualized using FigTree 1.4.2.

## Results

### Phylogenetic analysis

The ITS dataset included 11 sequences representing eight *Cystolepiota* species, and *Lepiota clypeolaria* was chosen as the out-group (Table 1). The alignment contained 695 characters, of which 434 characters were constant; 73 were parsimony uninformative; and 188 were parsimony informative. Gaps were treated as missing data. One most parsimony tree was found in the heuristic search. The tree has a CI of 0.799, RI of 0.761, RC of 0.608, HI of 0.201, and TL of 442. GTR+G was selected as the best model by hierarchical likelihood ratio tests (hLRTs) in MrModeltest 2.3 through using the Akaike information criterion. The average deviation of split frequencies at the end of the run was 0.003267.

**Table 1.** Voucher table.

Taxon	GenBank accession number
*Cystolepiota hetieri*	JF907982
*Cystolepiota hetieri*	AY176459
*Cystolepiota bucknallii*	AY176458
*Cystolepiota adulterina*	JF907978
*Cystolepiota pulverulenta*	AF391037
*Cystolepiota pulverulenta*	AF391036
*Cystolepiota seminuda*	AY176350
*Cystolepiota fumosifolia*	EF121817
* Cystolepiota pseudofumosifolia HMAS276122 Holotype	KF804000
* Cystolepiota pseudofumosifolia HMAS276123	KF804001
*Lepiota clypeolaria*	JN944094

*The sequences that were produced by this research.

**Figure 1.**
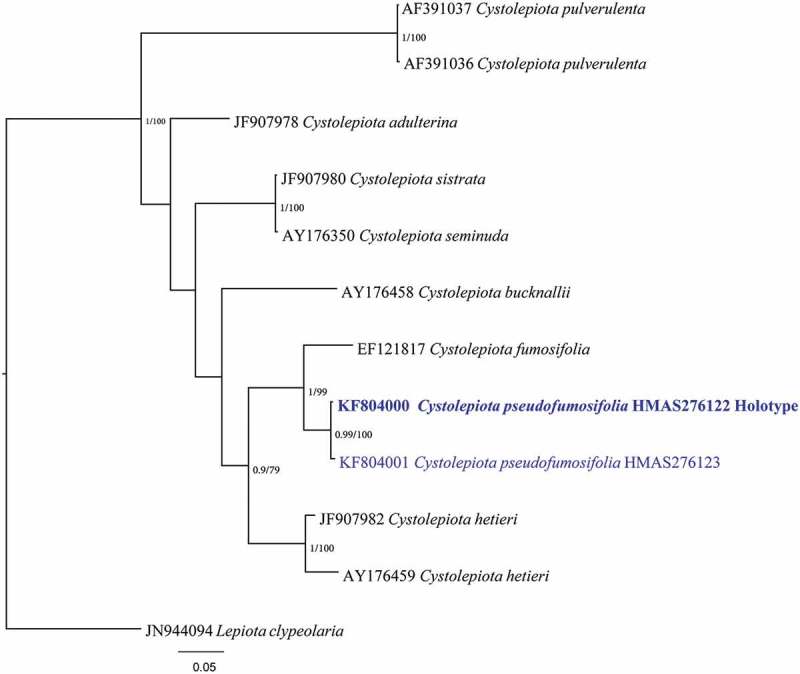
Phylogeny of *Cystolepiota* generated from a Bayesian analysis of ITS sequences, rooted by *Lepiota clypeolaria*. Bayesian posterior probability (PP) and maximum parsimony bootstrap support (BS) >0.95 and 75, respectively, are given at the internodes (PP/BS). Holotype of the new taxa is in bold.

The phylogenetic trees estimated by MP and Bayesian inference had almost identical topologies. The Bayesian tree was presented (Figure 1). The two samples from this new species grouped together with strong support (BS = 100, PP = 0.99) and appeared sister to the subclade of *Cystolepiota fumosifolia* (BS = 99, PP = 1; Figure 1).

## Taxonomy

**Figure 2.**
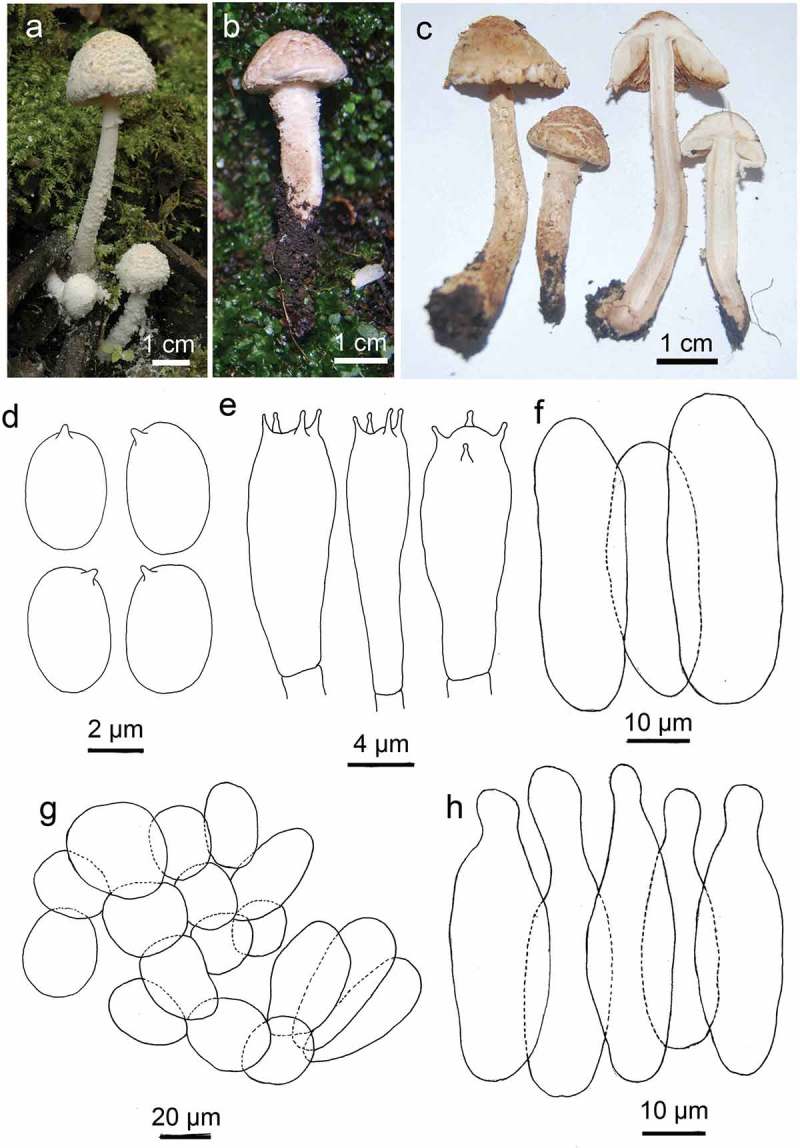
*Cystolepiota pseudofumosifolia* sp. nov. (a. and b. from specimen HMAS276123, c. from specimen HMAS276122, Holotype); d. basidiospores (scale bar = 2 μm); e. basidia (scale bar = 4 μm); f. marginal cells (scale bar = 10 μm); g. pileipellis cells (scale bar = 20 μm); h. cheilocystidia (scale bar = 10 μm).

*Cystolepiota pseudofumosifolia* M.L. Xu & R.L. Zhao, sp. nov. (Figure 2).

Fungal names: FN570270

Etymology: refers to the similarity to the species *C*. *fumosifolia* (Murrill) Vellinga in morphology.

Pileus 15–22 mm in diameter, hemispherical, and with densely floccose-verrucose covering when young, later expanding to conic, wide conic, surface heavily granulose to powdery, and often forming thick squamose, somewhat cracked, pitch-like, light brown (oac765), brown (oac647) on a whitish background when old, margin straight. Context firm, white, 2- to 3-mm thick at disc. Lamellae free, lamellulae in three series, close to crowded, ventricose, 4-mm broad, white, edges smooth. Stipe 30–70 × 4–8 mm, cylindrical or long clavate, hollow, surface light yellowish brown, brown, heavily squamose, warted, or powdery. Annulus membranous, smooth at the upper side and floccose at lower side, white, broken. No discolouring on touching or bruising.

Basidiospores 4.2–5.2 × 1.9–2.5 μm, avl × avw = 4.9 × 2.2 μm, Q = 1.8–2.6, avQ = 2.2, ellipsoid to oblong, rarely cylindrical, smooth, hyaline. Basidia 19–21 × 4.2–6.5 μm, four-spored, smooth, and clavate. Cheilocystidia numerous, ventricose-capitate, hyaline, the width reaching 18 μm, and the length reaching 40 μm. Pleurocystidia not observed. Marginal cells separated with each other, 43–52 × 9–18 μm, smooth, yellow, and cylindrical without ventricose-capitate. Pileipellis epithelium, composed of loosely globose and elliposoid cells in chains, 34–60 × 24–40 μm, and contains yellow pigments. Clamp connect present.

Habit: solitary or scattered in deciduous woods on nutrient-rich soil.

Material examined: China, Yunnan Prov., Shizong County, Yingwu Mountain, 29 June 2011, Collector Ruilin Zhao, *ZRL2011054* (HMAS276122, Holotype); Yunnan Prov., Nanjian County, Wuliang Mountain National Natural Reserve, 3 July 2012, Collector Ruilin Zhao, *ZRL2012038* (HMAS 276123).

## Discussion

Vellinga provided an in-depth and comprehensive description of *Cystolepiota*, including a key to *Cystolepiota* species (Vellinga 2001, 2007). *Cystolepiota bucknallii* (Berk. & Broome) Singer & Clémençon and *Cystolepiotaicterina* F.H. Møller ex Knudsen have lilac-tinged pileus; the cap of *Cystolepiotamoelleri* Knudsen is covered with small pink granulose warts, and *C. seminuda* (Lasch) Bon has a vinaceous stipe, characters which make them easily differentiated from *C. pseudofumosifolia* in the field. *Cystolepiota petasiformi*s (Murrill) Vellinga and *Cystolepiotapulverulenta* (Huijsman) Vellinga are distinct species in this genus because they have elongate and inflated pileipellis cells, which is also an obvious feature to separate it from the new species. *Cystolepiota oregonensis* (H.V. Sm.) Vellinga is similar to the new species in the field, but the former shows reddish-brown discoloration after touching or bruising.

The phylogenetic tree of *Cystolepiota* indicated that our samples belong to *Cystolepiota* and represented a different species from *C. bucknallii, Cystolepiota adulterina* (F.H. Møller) Bon, *C. seminuda, C. hetieri*, and *C. pulverulenta* (Huijsman) Vellinga (Figure 1). Furthermore, these two specimens belong to the same species and are closely related to *C. fumosifolia* (Murrill) Vellinga. *C*. *pseudofumosifolia* is also morphologically similar to *C. fumosifolia* because they share not only macroscopic characters but also microscopic characters, such as size and shape of the pileipellis and cheilocystidia (Vellinga 2006). However, our species presents smaller and narrower spores than those of *C. fumosifolia* (4.9–5.7 × 2.6–3.1 μm, avQ = 1.8). A key to known species of *Cystolepiota* in China is provided.

**Key to the known species of *Cystolepiota* in China**
1. Length of part or all of the basidiospores over 4 μm2
1. Length of all of the basidiospores not over 4 μm3
2. Lamellae adnate, pileus up to 4 cm *Cystolepiota lignicola*
2. Lamellae free, pileus up to 3 cm or less3
3. Pileus white with a flesh-colour tinge. Stipes with pinkish tinge towards the base. Context white with pinkish tinge in lower stem *Cystolepiota sistrata*
3. Pileus whitish tinged. Stipes dark red when bruised. Context white *C. seminuda*
4. Lamellae cream, stipes white, with vinaceous towards the base of the stem *Cystolepiota adulterine*
4. Lamellae white, stipes white, with brownish tinges 5
5. Stipes turning reddish-brown tinge when touched *C. hetieri*
5. None of the above 6
6. Stipes white with dusty brown tinge *C. pseudogranulosa*
6. Stipes white with yellowish brown tinge *C. pseudofumosifolia*
